# The Therapeutic Use and Potential of MSCs: Advances in Regenerative Medicine

**DOI:** 10.3390/ijms26073084

**Published:** 2025-03-27

**Authors:** Alin Constantin Pînzariu, Roxana Moscalu, Radu Petru Soroceanu, Minela Aida Maranduca, Ilie Cristian Drochioi, Vlad Ionut Vlasceanu, Sergiu Timofeiov, Daniel Vasile Timofte, Bogdan Huzum, Mihaela Moscalu, Dragomir Nicolae Serban, Ionela Lacramioara Serban

**Affiliations:** 1 Department of Morpho-Functional Sciences II, Discipline of Physiology, “Grigore T. Popa” University of Medicine and Pharmacy, 700115 Iasi, Romania; alin.pinzariu@umfiasi.ro (A.C.P.); minela.maranduca@umfiasi.ro (M.A.M.); dragomir.serban@umfiasi.ro (D.N.S.); ionela.serban@umfiasi.ro (I.L.S.); 2 Division of Cell Matrix Biology & Regenerative Medicine, School of Biological Sciences, Faculty of Biology, Medicine and Health, The University of Manchester, Manchester M13 9PL, UK; 3 Department of Surgery I, Discipline of Surgical Semiology, “Grigore T. Popa” University of Medicine and Pharmacy, 700115 Iasi, Romania; vlad-ionut.vlasceanu@umfiasi.ro (V.I.V.); stimof@yahoo.com (S.T.); daniel.timofte@umfiasi.ro (D.V.T.); 4 Department of Oral and Maxillo Facial Surgery, “Grigore T. Popa” University of Medicine and Pharmacy, 700115 Iasi, Romania; ilie-cristian.drochioi@umfiasi.ro; 5 Department of Orthopaedic and Traumatology, “Grigore T. Popa” University of Medicine and Pharmacy, 700115 Iasi, Romania; bogdan.huzum@umfiasi.ro; 6 Department of Preventive Medicine and Interdisciplinarity, “Grigore T. Popa” University of Medicine and Pharmacy, 700115 Iasi, Romania; moscalu.mihaela@gmail.com

**Keywords:** mesenchymal stem cells, regenerative medicine, tissue regeneration, immunomodulation, stem cell therapy

## Abstract

Mesenchymal stem cells (MSCs) have emerged as a relevant strategy in regenerative medicine due to their multipotent differentiation capacity, immunomodulatory properties, and therapeutic applications in various medical fields. This review explores the therapeutic use of MSCs, focusing on their role in treating autoimmune disorders and neoplastic diseases and in tissue regeneration. We discuss the mechanisms underlying MSC-mediated tissue repair, including their paracrine activity, migration to injury sites, and interaction with the immune system. Advances in cellular therapies such as genome engineering and MSC-derived exosome treatments further enhance their applicability. Key methodologies analyzed include genomic studies, next-generation sequencing (NGS), and bioinformatics approaches to optimize MSC-based interventions. Additionally, we reviewed preclinical and clinical evidence demonstrating the therapeutic potential of MSCs in conditions such as graft-versus-host disease, osteoarthritis, liver cirrhosis, and neurodegenerative disorders. While promising, challenges remain regarding standardization, long-term safety, and potential tumorigenic risks associated with MSC therapy. Future research should focus on refining MSC-based treatments to enhance efficacy and minimize risks. This review underscores the need for large-scale clinical trials to validate MSC-based interventions and fully harness their therapeutic potential.

## 1. Introduction

**Figure 1:**
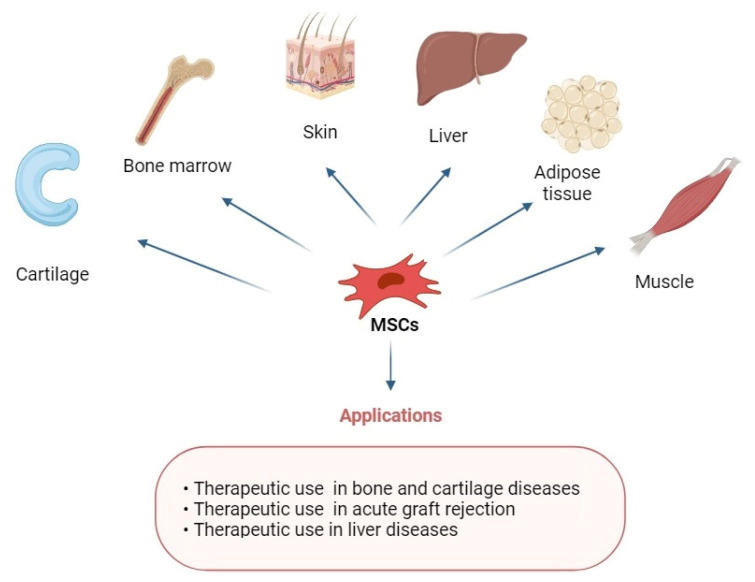
Applications of MSCs with multiple differentiation potential for repair of various tissues.

Mesenchymal stem cells (MSCs) have garnered increasing interest due to their remarkable applicability in a vast range of clinical challenges, from their key role in supporting tissue regeneration and wound healing to more immunomodulatory effects [1]. MSCs are multipotent stem cells able to self-renew and easily proliferate and can be widely derived from bone marrow, adipose tissue, placentas, umbilical cords, skin, connective muscle tissue, the liver, and the spleen, amongst other sources [2]. The inherent ability of MSCs to undergo differentiation into a wide array of cell lineages including osteoblasts and chondrocytes, adipocytes, and neuronal or muscle cells [3,4,5], and their accessible in vitro expansion and low ethical impact [2], have attracted considerable attention for their effective utilization in the realm of therapeutic strategies for various human diseases (Figure 1).

The literature has described their role in tissue regeneration through their unique ability to migrate to the site of injury, their paracrine potential, their ability to release growth factors and cytokines [6], and their low immunogenic effects, allowing for easier allogeneic transplants that do not require immunosuppressive therapies [2]. Upon transplantation, they acquire immunosuppressive properties, adapt to the structure of the transplanted tissue, and subsequently advance into progenitor cells within the microenvironment, functioning both as stem cells and their progeny [7]. They possess natural abilities to detect changes in their environment, such as the appearance of inflammation. Under such conditions, they can induce the release of bioactive agents and form progenitor cells in response to these changes [8]. Additionally, it has been demonstrated that MSCs migrate to sites of inflammation far from their injection site [9,10].

As research into regenerative medicine and cellular therapies continues to progress, the use of MSCs is becoming increasingly recognized for its valuable therapeutic properties [9]. In most cases, these properties have a cumulative effect on the successful achievement of the desired biological effects of MSCs. However, their exact roles in mediating therapeutic actions are not yet fully understood [11].

In the clinical field, the therapeutic effects of MSCs are currently under investigation in clinical trials involving patients with diverse medical conditions. These conditions include orthopedic injuries, acute graft rejection following bone marrow transplantation, cardiovascular diseases, autoimmune disorders, and liver diseases. Moreover, the genetic engineering of MSCs to enhance the expression of antitumor genes has opened up promising perspectives for their potential clinical application as an antineoplastic therapy [11].

The first clinical study using MSCs developed ex vivo was conducted in 1995 by Lazarus et al. [12]. During this Phase I feasibility study, 15 patients with hematological malignancies successfully received autologous MSC injections without any toxic adverse effects [11,12]. Subsequent to this initial study, many clinical investigations have been undertaken to assess the viability and effectiveness of MSC therapy across diverse pathological conditions. These studies primarily fall within Phase I, which evaluates safety; Phase II, focused on establishing proof of concept and efficacy in human subjects; or a combination of both (Phase I/II). Only a limited proportion of these clinical trials have advanced to Phase III, where newer treatments are compared to standard therapies or the most established methods of therapy or involve a combination of Phase II/III studies [11].

In general, MSCs exhibit a favorable safety profile, with most investigations indicating a lack of medium-term adverse effects, as evidenced by the majority of investigations indicating the absence of significant medium-term adverse reactions. Nevertheless, a few studies have reported the occurrence of mild and transient peri-injection effects, which are generally short-lived and not of substantial concern [8]. Furthermore, many completed clinical studies have demonstrated the effectiveness of MSC infusions for various other diseases, such as acute myocardial ischemia, stroke, liver cirrhosis, amyotrophic lateral sclerosis, and osteoarticular diseases [11].

In the present review, we will discuss the diverse successful utilizations of MSCs to address a wide variety of common important global clinical challenges, including solid organ transplant rejection and graft-versus-host disease; bone and cartilage disease, including osteoarthritis and osteogenesis imperfect; liver diseases; autoimmune conditions, with emphasis on systemic lupus erythematosus, multiple sclerosis, and rheumatoid arthritis; and neoplastic diseases. We will also investigate the immunomodulatory mechanisms of MSCs that improve graft survival and reduce inflammation, promoting tissue regeneration in conditions such as osteoarthritis or cirrhosis, and examine developing targeted therapies for cancer that use the ability of MSCs to deliver anticancer agents. This review highlights the versatility of MSC therapies and the potential for further development of therapeutic strategies that can benefit from their vast availability, ease of culturing ex vivo, and safe integration, whether in vivo or in vitro.

### Highlights from the Specialized Literature Regarding the Therapeutic Use and Potential of MSCs

The literature search was conducted in the PubMed database (accessed on 1 February 2025), focusing on studies published within the last 10 years. Our search queried “mesenchymal stem cells [AND] regenerative medicine [OR] tissue regeneration [OR]; immunomodulation [AND] stem cell therapy” and was limited only to prospective and retrospective studies and metaanalyses, omitting abstracts, documents, and reviews. In this way, our search resulted in 48 references within our scope of interest, covering the period from 2015 to 2025, of which 20 articles were identified in the last five years. Additionally, the bibliographic references of the identified studies were examined, and specific observations relevant to the objectives of our study were integrated. Significant viewpoints consistent with our research are included in the discussion section, leading to the referencing of articles prior to 2015 when deemed relevant.

## 2. Therapeutic Use of MSCs in Acute Graft Rejection

The successful use of MSC therapy for a severe case of acute graft rejection refractory to steroid treatment in a nine-year-old leukemia patient following allogeneic hematopoietic stem cell transplantation (HSCT) has sparked increased interest in the research world [13,14]. This study led the way for further clinical trials and preclinical studies that thoroughly investigated the potential of MSCs not only in graft-versus-host disease (GVHD)-related HSCT but also for the treatment and prevention of solid organ transplant rejection [14]. Linked with significant morbidity and mortality rates, the current standard-of-care treatment for acute transplant rejection is immunosuppression using corticosteroids [15]. However, this therapy has demonstrated effectiveness only in a small subset of patients, leaving room for further improvement in treatment outcomes.

A follow-up study by Fang et al. [16] tested adipose tissue-derived MSCs in six patients with severe grade III-IV acute GVHD that did not respond to steroids. They found that five out of six—around 83%—had their GVHD resolve, with the intestine, liver, and skin completely healing in those cases. Monitoring these patients later showed they lived longer, anywhere from a few months to three years for some, though survival varied greatly from one person to the next [10,16,17,18,19].

The mechanism of action of MSC therapy in GVHD or solid organ transplantation rejection has been widely linked with their ability to inhibit the activity of effector T cells, which drive rejection by causing excessive transplanted tissue damage [20], and instead modulate the increase in anti-inflammatory regulatory T cells (Treg), which suppress the immune system, thus improving graft survival. This was also demonstrated in vivo in a murine semiallogeneic heart transplant model, which highlighted the immunosuppressive potential of MSC therapy by inducing donor-specific CD4+CD25+Foxp3+ Treg cells, key factors in preventing autoimmune disease and maintaining immune tolerance [21]. These effects are largely due to the paracrine activity of MSCs, secreting factors such as transforming growth factor beta (TGF-β), nitric oxide (NO)—which modulates to prevent T-cell division by interfering with their signals, prostaglandin E2 (PGE2)—which facilitates attenuation by cutting pro-inflammatory cytokines and directing macrophages to a subdued M2 type, or indoleamine 2,3-dioxygenase (IDO) as a response to the GVHD-related elevation of pro-inflammatory cytokines including interferon gamma (IFN-γ), tumor necrosis factor alpha (TNF-α), or interleukin 1β (IL-1β) [14]. The anti-inflammatory effects of MSCs have also been identified through their role in supporting the change in polarization of macrophages, from the pro-inflammatory M1 phenotype to a pro-healing M2 phenotype [22], further supporting a dampening of the inflammatory response and promoting tissue repair and graft survival.

MSC therapy has also shown promising results in improving graft survival and reducing the rate of transplant rejection in a number of other tissue transplants, including liver, kidney, cornea, and skin [14,23,24,25,26], and multiple clinical trials are underway to further address, test, and demonstrate the successful immunomodulatory effects of MSCs [14].

## 3. Therapeutic Use of MSCs in Bone and Cartilage Diseases

The multipotent capacity of MSCs and, specifically, their ease of differentiation into osteoblasts (bone-forming cells), tenocytes (tendon cells), and chondrocytes (cartilage cells) has garnered significant attention for their potential application in orthopedic conditions. MSCs have been used in the development of regenerative therapy strategies aimed at treating orthopedic injuries and promoting tissue repair, as well as regeneration in the musculoskeletal system [27,28,29].

MSCs have demonstrated a beneficial role in the treatment of bone system diseases, particularly conditions like osteogenesis imperfecta and hypophosphatasia. Osteogenesis imperfecta (OI) is a genetic disorder characterized by increased skeletal fragility and alterations in connective tissue due to the deficient production of type I collagen by osteoblasts [11]. Also, a genetic disorder, hypophosphatasia, is the result of a loss of function mutation in the alkaline phosphatase gene that encodes the activity of tissue-nonspecific alkaline phosphatase (TNSALP) [30]. These diseases are characterized by abnormalities in bone formation and mineralization, leading to brittle bones and skeletal deformities. MSCs differentiate into osteoblasts under the influence of signaling molecules such as bone morphogenetic proteins (BMPs) and Runx2, a transcription factor that initiates the whole bone-making process. Once they differentiate, they produce type I collagen to reinforce the structural deficiencies in OI’s bone setup and enhance alkaline phosphatase levels to address the impaired mineralization in hypophosphatasia [28,29,30]. Apart from the anti-inflammatory and immunomodulatory advantages brought about by MSC therapy, these cells have also been shown to contribute to bone regeneration by being able to migrate to the site of a bone injury due to specialized chemokine receptor activity [31], promote osteoblast differentiation, and localize new blood vessel formation, which is essential for bone repair [31,32,33].

A series of clinical studies focused on pediatric patients with OI who underwent allogeneic hematopoietic stem cell transplants has shown promising outcomes. The transplanted bone marrow cells successfully integrated into the recipient’s body and gave rise to functional osteoblasts, contributing to the enhancement of bone structure and overall function [34,35,36,37,38]. These findings offer encouraging prospects for the potential therapeutic use of hematopoietic stem cell transplantation in managing OI and promoting bone health in affected pediatric patients.

MSCs have also shown great therapeutic potential in the treatment of osteoarthritis (OA). OA has a high incidence worldwide, with a high socioeconomic and psychological impact due to persistent pain and disability that erode patients’ quality of life (QoL) [39,40]. For OA, MSCs undergo chondrogenic differentiation into chondrocytes when transforming growth factor-beta (TGF-β) and Sox9, a transcription factor serving as a pivotal regulator of chondrogenic differentiation and cartilage formation, which directs the production of cartilage matrix components—such as type II collagen and aggrecan—to repair cartilage degradation. They also release anti-inflammatory factors to mitigate the joint’s inflammatory disruption, helping repair it over time [41]. However, there is still a paucity of effective therapeutic strategies beyond the surgical replacement of the affected joint. Advances in MSCs’ regenerative therapies have brought about the successful integration of these cells for OA management. A meta-analysis by Tan et al. [41], comprising nine studies and 440 osteoarthritic knees, concluded that even in the absence of adjuvant therapies, intra-articular MSC injections significantly improved function and joint pain in affected individuals [41,42]. The results were also reflected throughout patient questionnaires, such as the Western Ontario and McMaster Universities Osteoarthritis Index (WOMAC) analyzing pain, stiffness, and knee function, and the Knee Injury and Osteoarthritis Outcome Score (KOOS) questionnaire looking into the impact of knee injuries on patients’ QoL [40,41].

These are just a few examples of the impact that MSC therapies can have in orthopedics, providing effective solutions to ongoing clinical challenges that are likely to increase in the modern population.

## 4. Therapeutic Use of MSCs in Liver Diseases

While MSCs have been closely studied in the aforementioned clinical contexts, the research to date shows that their use in treating hepatic cirrhosis has remained restricted to a narrowly defined patient group.

In a Phase I clinical trial conducted by Mohamadnejad et al. [42], four patients with decompensated hepatic cirrhosis were administered autologous MSCs intravenously. Throughout the study, no adverse effects were observed in the patients, and notably, their QoL exhibited a considerable improvement by the end of the study’s follow-up phase [43]. A deeper look into the results suggests that the boost in quality of life (QoL) stemmed from better liver performance, particularly marked by increased albumin levels, rather than MSCs turning into hepatocytes or endothelial cells—something this study did not convincingly show [43].

In a separate Phase I-II clinical trial, eight patients diagnosed with end-stage liver disease (comprising four with hepatitis B, one with hepatitis C, one with alcoholic hepatopathy, and two with cryptogenic etiology) were administered injectable autologous MSCs. The research demonstrated that all participants responded well to the therapy, with notable enhancements in liver function being particularly evident. These findings support the notion that MSCs may offer a feasible, safe, and efficient treatment option for individuals with end-stage liver diseases [44]. Through paracrine effects, MSCs seem to mitigate chronic inflammation—a key contributor to these disorders—by secreting anti-inflammatory factors like IL-10 and TGF-β, which reduce excessive immune responses, including overactive macrophages [44].

A systematic review and meta-analysis by Lu et al. [45] identified 11 randomized control clinical trials addressing the use of MSC strategies in liver cirrhosis between 2011 and 2023. These studies showed that MSC infusions were safe, with no presented adverse side effects, and had made a significant positive improvement on albumin levels from two weeks up to six months from infusion compared with the controls. Moreover, an increased three-month survival rate was reported based on the patients’ MELD scores at one, two, and six months post-treatment [45].

However, in the context of a progressive disease with debilitating factors that can significantly impact patients’ QoL, there is a paucity of studies that needs to be addressed before MSCs can routinely be used in liver disease. This mainly revolves around the effects of different sources of MSCs, optimization of dosages and routes of administration, as well as frequency of treatment [45,46].

## 5. Therapeutic Use of MSCs in Autoimmune Diseases

The literature data highlight attempts to use MSCs in the therapy of autoimmune conditions such as refractory cases of systemic lupus erythematosus (SLE), multiple sclerosis (MS), polymyositis, vasculitis (including Behcet’s disease), rheumatoid arthritis, Crohn’s disease, polychondritis, and encephalomyelitis [47,48,49,50].

Recent in vitro studies have revealed that MSCs possess immunomodulatory characteristics and can exert immunosuppressive effects on various immune cell types. These effects include the inhibition of lymphocyte proliferation, memory, and activated T cells, B cells, natural killer (NK) cells, and dendritic cells. It is important to differentiate this concept from that of autologous hematopoietic stem cell transplantation, as MSC transplantation does not necessitate immunosuppression of patients beforehand. Furthermore, the therapeutic impact of MSCs is considered to occur locally within the inflamed organ, facilitated by the engraftment of MSCs in that particular area. Consequently, the effects of MSC therapy are relatively short-lived, limited to the immediate vicinity of grafting [51,52].

SLE is a systemic chronic autoimmune and inflammatory disorder that can lead to multiorgan injury, including the brain, lungs, and kidneys, as well as the hematopoietic system [53]. Some researchers revealed that the transplantation of bone marrow-derived mesenchymal stem cells improved multi-organ function in genetically modified mice and individuals diagnosed with SLE. The genetically modified mice (MRL/lpr) were found to have a deficiency in the osteoblastic niche, which seems to be partially responsible for the pathogenesis of systemic lupus erythematosus [54,55,56]. Moreover, the allogeneic transplantation of bone marrow-derived MSCs has the capability to rebuild the bone marrow osteoblastic niche and shows enhanced effectiveness in reducing multi-organ dysfunction when compared to cyclophosphamide, the conventional standard therapy. Patients with SLE who received allogenic bone marrow-derived mesenchymal stem cell transplants experienced stable disease remission for a period ranging from 12 to 18 months. Additionally, improvements in symptoms like joint pain and fatigue, alongside decreasing serological markers and better renal function, were seen [57,58]. Other experimental research has demonstrated favorable effects of MSC use in the treatment of multiple sclerosis, but the mechanisms underlying these actions are not fully elucidated, suggesting possible involvement in immunomodulation, neuroprotection, and neuro-regeneration as shown in (Table 1) [59,60,61,62].

The use of MSC strategies for patients with MS, an autoimmune neurodegenerative disease causing localized inflammation, demyelination, and axon degeneration within the central nervous system [63], has also been investigated. A systematic review and meta-analysis by Vaheb et al. [64], updated in January 2024 and covering 30 studies, examined the safety and efficacy of this cell therapy in patients with various types of MS, including relapsing-remitting, primary, and secondary progressive disease. Interestingly, the intrathecal (IT) approach demonstrated significantly higher improvements in patients’ expanded disability status scale (EDSS) when compared with the control, rather than the intravenous (IV) approach of administering the MSCs. This could be due to the limited ability of IV-administered MSCs to cross the blood–brain barrier, which hinders their therapeutic effects [64,65]. MRI imaging has also highlighted that 71.8% of patients had activity-free MRI scans in short-term follow-up and 100% clearance with no Gadolinium-enhancement lesions (GELs) on long-term follow-up scans [64]. This confirms MSCs’ role in plaque remyelination, as repeated exposure through IT MSC infiltrations for a prolonged duration has been shown to promote the secretion of trophic factors, including leukemia inhibitory factor (LIF), insulin-like growth factors (IGF), glial cell-line-derived neutrophic factor (GDNF), and hepatocyte growth factor (HGF), which can potentiate the neural repair in the context of plaque demyelination [64,66,67,68] and also dampen the localized inflammation, thus inhibiting further development of GELs [64,69,70].

**Table 1:** MSC therapy research.

Study	Study Type	Therapy	Targeted Condition	Mechanism of Action	Main Findings
Islam et al., 2023 [62]	Meta- analysis	MSC therapy for multiple sclerosis	Multiple Sclerosis	Neuroprotection, reducing relapses and disability	Meta-analysis confirms MSCs’ potential in reducing relapses and disability in MS patients.
Vaheb et al., 2024 [63]	Meta- analysis	MSCs’ neurological efficacy in MS	Multiple Sclerosis	Enhancing neurological function, reducing progression	Updated review highlights the safety and efficacy of MSCs in progressive MS therapy.
Terstappen et al., 2021 [64]	Review	Therapeutics across the blood–brain barrier	Blood–Brain Barrier	Improving MSC delivery for CNS repair	Discusses methods to improve MSC delivery to CNS via blood–brain barrier crossing.
Harris et al., 2012 [65]	Preclinical study	Autologous MSC-derived neural progenitors	Multiple Sclerosis	Using MSC-derived neural progenitors for CNS regeneration	Demonstrates feasibility of MSC-derived neural progenitors for CNS applications.
Huang and Dreyfus, 2016 [67]	Review	Growth factors for demyelinating diseases	Demyelinating Diseases	Using growth factors to support remyelination	Growth factors like IGF and FGF can support MSC-based therapies in demyelinating diseases.
Petrou et al., 2020 [68]	Clinical trial	MSC transplantation for progressive MS	Progressive Multiple Sclerosis	Autologous MSC transplantation for neurological repair	Clinical benefits observed in MS patients post MSC transplantation, improving neurological function.
Harris et al., 2020 [69]	Clinical trial	MSC-derived neural progenitors for MS	Progressive Multiple Sclerosis	Long-term benefits of MSC-derived neural progenitors	Phase I study indicates long-term benefits of MSC-derived progenitors in progressive MS.
Zhang et al., 2019 [71]	Preclinical study	Different MSCs for rheumatoid arthritis	Rheumatoid Arthritis	Comparison of MSC types for immune regulation	Comparison of different MSC sources shows varying efficacy in RA models.
Hwang et al., 2021 [72]	Review	Clinical applications of MSCs in RA and OA	Rheumatoid Arthritis and Osteoarthritis	Reduction of inflammation and joint damage	Recent trials confirm the effectiveness of MSCs in reducing inflammation in RA and OA.
Wang et al., 2013 [11]	Clinical trial (Phase II)	Umbilical cord MSCs for rheumatoid arthritis	Rheumatoid Arthritis	Umbilical cord MSCs modulating immune response	Human umbilical cord MSCs show good safety and efficacy profile for RA patients.
Park et al., 2018 [73]	Clinical trial (Phase I)	Intravenous MSCs for RA (Phase 1 trial)	Rheumatoid Arthritis	MSC infusion reducing symptoms in RA patients	Phase 1 clinical trial supports intravenous MSC administration as a viable RA treatment.
Ghoryani et al., 2020 [74]	Clinical trial	Immunoregulatory effects of MSCs in RA	Rheumatoid Arthritis	Enhancing regulatory T cells for immune suppression	MSC therapy modulates immune response, increasing regulatory T cells in refractory RA.

Treatment strategies involving MSCs have also been developed for rheumatoid arthritis (RA), an autoimmune condition affecting patients’ joints, muscles, and tendons, as well as connective and fibrous tissue, leading to severe and debilitating joint deformities, dysfunction, and pain [71]. Although a much more limited number of studies have been presented in the specialized literature, the safety and therapeutic effects of MSC administration have been highlighted in in vivo mice models [72]. Clinical trials have also been developed. Wang et al. [14] also confirmed the safe administration of MSCs in patients with RA and disease-modifying anti-rheumatic drugs (DMARDS) and showed improvements in disease activity based on a disease activity score of 28 (DAS28), joint swelling and pain, and overall QoL [14,72]. Another clinical trial by Park et al. [73] also noted a dose-dependent decrease in DAS28 and reported no severe adverse effects following MSC therapy; apart from a mild elevation of serum uric acid levels, no other changes were noted in the hematological or chemical investigations of patients [71,72]. Ghoryani et al. [74] went further, highlighting the immunomodulatory effects of MSC therapy in refractory RA, identifying a post-infiltration elevation of IL-10, TGF-β, and head box P3 (FOXP3), a protein regulating the development and function of Treg cells [72,74].

It is clear that there is still space for the development of MSC-targeted strategies for auto-immune disorders. Previous studies have provided the basis of ensuring safety and efficacy in human applications of MSCs; however, considering the complexity of these disorders and their systemic effects, there is a high need for large, comprehensive studies that can inform the decision-making and guide the adjustment of MSC therapies for the affected population.

## 6. Therapeutic Use of MSCs in Neoplastic Diseases

Although preclinical models extensively explored the utilization of genetically modified MSCs for treating diverse cancer types such as breast, lung, liver, pancreatic, ovarian, prostate, sarcoma, and glioma, clinical trials implementing MSCs in neoplastic disease therapy have yet to be documented [75]. While no significant adverse events have been observed in their application, the safety of MSC administration remains a current area of concern.

The potential of MSCs to undergo malignant transformation is a significant concern that poses limitations on ensuring their safety in therapeutic applications [76]. The tumor microenvironment plays a crucial role in influencing tumor-cell behavior, creating an atmosphere that can either promote or suppress tumor development. MSCs have demonstrated the ability to modulate the fate of tumor cells, attracting attention to their potential utility in this context. MSCs are capable of integrating into the tumor microenvironment, a region abundant in inflammatory mediators, cytokines, and growth factors. This setting may prompt MSCs to secrete agents that amplify angiogenesis, cellular proliferation, and tumor invasion, thereby potentially aggravating tumor expansion and metastatic spread. For instance, the liberation of vascular endothelial growth factor (VEGF), a key angiogenic promoter, or other proliferative cues could enhance vascular support and drive tumor growth within this context [77].

MSCs have been acknowledged for their dual effects, encompassing both tumorigenic and antitumoral activity. On the one hand, clinical studies have shown promising potential for MSCs in the treatment of human cancer. Notably, MSCs possess intrinsic migratory abilities, which hold promise for targeted drug delivery and therapies aimed at tumor and metastatic cells. Moreover, different manipulation techniques have been explored to achieve the desired expression of anti-angiogenic, anti-proliferative, and pro-apoptotic properties tailored to specific tumor types [78]. For instance, liberating vascular endothelial growth factor (VEGF), a key angiogenic promoter, or other proliferative cues could enhance vascular support and drive tumor growth within this context [75,79].

Tumor angiogenesis, a critical feature of tumor advancement and metastasis, has garnered significant attention as a therapeutic target in cancer treatment since the introduction of the first angiogenesis inhibitor, bevacizumab, for metastatic colorectal cancer therapy. MSCs demonstrate pathotropic migratory attributes, rendering them promising con-tenders for the targeted delivery of antineoplastic agents. Given the advancements in specific anticancer drugs and considering the incorporation and targeted delivery capabilities of MSCs, a novel area of research has emerged aiming to develop efficacious cancer therapies by manipulating these cells [80].

The scientific literature provides evidence that unmodified MSCs exhibit antitumor effects in vitro and in various mouse tumor models. These effects are attributed, in part, to the release of factors by MSCs that possess antitumor functions, leading to reduced proliferation of melanoma, glioma, lung cancer, hepatoma, and breast adenocarcinoma cells [1]. It has been highlighted that bone marrow-derived MSCs injected intravenously into a mouse Kaposi’s sarcoma model colonize tumor sites and strongly inhibit tumor development [81].

Furthermore, experimental observations have demonstrated that MSCs display anti-angiogenic properties in both in vitro investigations and a murine model of melanoma. Moreover, when MSCs are directly injected into murine subcutaneous melanoma, it induces apoptosis and leads to a noteworthy reduction in tumor growth. In separate investigations, MSCs derived from umbilical cord blood have been utilized as untainted cellular entities in therapeutic approaches for treating multiform glioblastoma [7].

Stem cells derived from umbilical cord blood, exhibiting elevated levels of CD44 and CD133, undergo apoptosis upon co-culture with multiform glioblastoma cells. Additionally, the application of umbilical cord blood-derived stem cells in the treatment of glioma cells exerts an inhibitory effect on angiogenesis mediated by focal adhesion kinase (FAK), resulting in the upregulation of homologous phosphatase and tension in glioma cells, as well as the downregulation of Akt and PI3K signaling molecules. Consequently, this inhibits migration and characteristic cellular lesion healing in glioma [7]. Furthermore, favorable outcomes have been achieved by modifying MSCs to overexpress specific target genes and express or secrete molecules such as interleukins, on the one hand, and additionally, interferons, pro-apoptotic proteins, and anti-angiogenic agents on the other hand. This selective modulation exerts beneficial effects, making targeted therapy a viable option for various types of cancer [7].

Only a few clinical trials have been reported on cancer therapy using MSCs. The initial clinical trial (Phase I/II), referred to as TREAT-ME1, demonstrated promising outcomes by employing genetically changed autologous mesenchymal stromal cells as a delivery vector for herpes simplex virus thymidine kinase (HSV-TK) to treat advanced gastrointestinal tumors [81,82].

Two additional clinical trials concentrated on MSC usage in ovarian cancer. The first study (Phase I/II) aimed to determine adverse effects and establish the appropriate dose of MSC transfected with oncolytic measles virus encoding NIS (MV-NIS) while evaluating its effectiveness in patients with ovarian cancer [81]. Another Phase I clinical trial employed human MSCs transfected with IFN-β to evaluate its safety and ascertain a well-tolerated dosage for treating ovarian cancer patients.

In an alternative Phase I clinical study, the safety and potential benefits of allogeneic bone marrow-derived MSCs in patients with prostate cancer were investigated. The investigations showed that MSCs were not efficiently targeted to tumor sites to exert therapeutic effects [82]. These studies were conducted with the aim of assessing the potential of MSCs in treating different types of cancer; however, the results appear to be insufficient and require additional preclinical studies and clinical trials.

## 7. Conclusions

In conclusion, MSCs hold great potential for regenerative medicine and therapeutic applications, making them effective in treating a wide range of medical conditions, including acute graft rejection, bone and cartilage diseases, liver diseases, autoimmune diseases, and select neoplastic diseases. Their ability to differentiate into various cell types, secrete bioactive molecules, and exhibit immunomodulatory properties makes them effective in treating a wide range of medical conditions, thereby facilitating tissue repair and improving damaged tissue functions. However, the research also underscores the importance of further investigations to establish comprehensive safety and efficacy profiles and to refine treatment methodologies. If, for some areas such as cardiovascular diseases, orthopedic diseases, or transplant rejection and graft-versus-host disease, the literature is more extensive and offers vast alternatives of treatment strategies for both the pediatric and adult populations, other areas, such as autoimmune disorders, neoplastic or solid organ diseases (i.e., liver, kidney, lung, etc.), still present a paucity of information regarding the efficacy and best administration strategies for achieving the desired results. As cellular therapies continue to advance in the field of regenerative medicine, MSCs remain a leading contender, offering hope for improved treatments and better patient outcomes in some of the biggest clinical challenges worldwide.
